# Optimization of the Sb_2_S_3_ Shell Thickness in ZnO Nanowire-Based Extremely Thin Absorber Solar Cells

**DOI:** 10.3390/nano12020198

**Published:** 2022-01-07

**Authors:** Guislain Hector, Jako S. Eensalu, Atanas Katerski, Hervé Roussel, Odette Chaix-Pluchery, Estelle Appert, Fabrice Donatini, Ilona Oja Acik, Erki Kärber, Vincent Consonni

**Affiliations:** 1 Université Grenoble Alpes, CNRS, Grenoble INP, LMGP, F-38000 Grenoble, France; guislain.hector@grenoble-inp.fr (G.H.); herve.roussel@grenoble-inp.fr (H.R.); odette.chaix@grenoble-inp.fr (O.C.-P.); estelle.appert@grenoble-inp.fr (E.A.); 2 Laboratory of Thin Film Chemical Technologies, Department of Materials and Environmental Technology, School of Engineering, Tallinn University of Technology, Ehitajate tee 5, 19086 Tallinn, Estonia; jako.eensalu@taltech.ee (J.S.E.); atanas.katerski@taltech.ee (A.K.); ilona.oja@taltech.ee (I.O.A.); 3 Université Grenoble Alpes, CNRS, Grenoble INP, Institut NEEL, F-38000 Grenoble, France; fabrice.donatini@neel.cnrs.fr

**Keywords:** ZnO nanowires, Sb_2_S_3_, chemical spray pyrolysis, core shell heterostructures, extremely thin absorbers, solar cells

## Abstract

Extremely thin absorber (ETA) solar cells made of ZnO/TiO_2_/Sb_2_S_3_ core–shell nanowire heterostructures, using P3HT as the hole-transporting material (HTM), are of high interest to surpass solar cell efficiencies of their planar counterpart at lower material cost. However, no dimensional optimization has been addressed in detail, as it raises material and technological critical issues. In this study, the thickness of the Sb_2_S_3_ shell grown by chemical spray pyrolysis is tuned from a couple of nanometers to several tens of nanometers, while switching from a partially to a fully crystallized shell. The Sb_2_S_3_ shell is highly pure, and the unwanted Sb_2_O_3_ phase was not formed. The low end of the thickness is limited by challenges in the crystallization of the Sb_2_S_3_ shell, as it is amorphous at nanoscale dimensions, resulting in the low optical absorption of visible photons. In contrast, the high end of the thickness is limited by the increased density of defects in the bulk of the Sb_2_S_3_ shell, degrading charge carrier dynamics, and by the incomplete immersion of the P3HT in the structure, resulting in the poor hole collection. The best ETA solar cell with a short-circuit current density of 12.1 mA/cm^2^, an open-circuit voltage of 502 mV, and a photovoltaic conversion efficiency of 2.83% is obtained for an intermediate thickness of the Sb_2_S_3_ shell. These findings highlight that the incorporation of both the absorber shell and HTM in the core–shell heterostructures relies on the spacing between individual nanowires. They further elaborate the intricate nature of the dimensional optimization of an ETA cell, as it requires a fine-balanced holistic approach to correlate all the dimensions of all the components in the heterostructures.

## 1. Introduction

Owing to its abundancy, non-toxicity, and relative ease to be grown as nanostructures by low-cost, low-temperature and easily implemented chemical deposition techniques [1,2], ZnO nanowires (NWs) have emerged as an important building block in nanostructured solar cells [3]. In particular, thanks to its high electron mobility as compared to TiO_2_ nanoparticles [4,5], ZnO NWs can act as an efficient electron transporting material (ETM) in a range of photovoltaic cells, including the so-called extremely thin absorber (ETA) solar cells [3,6,7,8,9]. In the core–shell configuration used in ETA solar cells, the n-type ZnO NWs are basically coated with a thin shell as the optical absorber in the visible part of the electromagnetic spectrum (hereinafter shell) [3]. The shell is typically an inorganic p-type semiconductor, exhibiting an electronic band structure with a direct bandgap energy ranging from 1.3 to 2.0 eV and a type II band alignment with ZnO NWs [3]. These core–shell heterostructures benefit from a large number of assets, including (i) efficient light absorption phenomena through radiated and guided optical modes and (ii) efficient charge carrier management through charge carrier separation and collection [10,11,12,13]. The first efficient ETA solar cell integrating ZnO NWs was reported in 2005 with the use of a p-type CdSe shell and of CuSCN as the hole transporting material (HTM), leading to the photovoltaic conversion efficiency (PCE) of 2.3% [14]. A wide variety of shells, including CdTe [15,16,17], CdSe [14,18,19], CdS [20], CdS/CdTe [21], In_2_S_3_ [22,23,24], TiO_2_/CuInSe_2_ [25,26], and Cu_2_O [27,28], as well as the wide bandgap ZnSe [29] and ZnS [30], through the type II interfacial transition, have been developed in ETA solar cells, along with different HTMs, such as CuSCN or iodide/triiodide and poly-sulfur electrolytes, resulting in PCEs lying in the range of 1.5–5%.

Following pioneering works in 1990s [31,32], antimony tri-sulfide (Sb_2_S_3_) as a p-type V/VI semiconductor with a high optical absorption coefficient of 7.5 × 10^4^ cm^−1^ at 550 nm and a direct bandgap energy of 1.7 eV at room temperature [33,34] has recently emerged as a highly promising optical absorber in semiconductor-sensitized solar cells [35,36,37]. The combination of Sb_2_S_3_ with nanoporous/mesoporous TiO_2_ has led to the fabrication of semiconductor-sensitized solar cells with a record PCE of 7.5% [38], following the optimization of HTMs and post-deposition treatments [39,40,41,42,43]. A special emphasis has recently been placed on the defect reduction and interface optimization to boost the fairly low open-circuit voltage (*V*_OC_) in Sb_2_S_3_-sensitized solar cells [44]. Alternative approaches for increasing efficiencies have included the development of Sb_2_(S,Se)_3_-sensitized solar cells, using in situ hydrothermal growth/post-selenization [45] or vapor transport deposition [46], leading to a PCE of 6.14 and 7.31%, respectively.

The combination of a Sb_2_S_3_ shell with TiO_2_ nanostructures in the form of nanofibers [47], nanotubes [48], or NWs [49,50,51,52] has also been explored, resulting in the fabrication of semiconductor-sensitized solar cells with a PCE in the typical range of 2–5%. Recently, the use of surface modifiers with different functional groups and carbon numbers has led to the fabrication of Sb_2_S_3_-sensitized solar cells, reaching a PCE of 5.37%, which showed the capability of getting a high photovoltaic performance, using oxide nanorods [52]. Alternatively, there has been an increasing interest in coupling an Sb_2_S_3_ shell with ZnO NWs [53,54,55,56,57,58,59,60]. First, ZnO NWs with a very short length of 100 nm have been gap-filled by Sb_2_S_3_, using vacuum evaporation, leading to the fabrication of a nanocomposite cell with a PCE of 2.9% [53]. An immersion into thioacetamide, followed by a metal cation exchange process, was further employed to transform the surface layers of ZnO NWs into ZnS/Sb_2_S_3_ shells, resulting in the fabrication of ETA solar cells with a PCE of 1.32% [54]. Later on, Parize et al. reported the first ETA solar cells integrating ZnO/TiO_2_/Sb_2_S_3_ core–shell NW heterostructures by using chemical bath deposition (CBD), atomic layer deposition (ALD), and ultrasonic chemical spray pyrolysis (CSP), respectively [55]. The TiO_2_ conformal shell with the anatase phase was used to act as a protective, passivating layer [61]. A PCE of 2.3%, along with a *V*_OC_ of 656 mV and a short-circuit current density (*J*_SC_) of 7.5 mA/cm^2^, was achieved by using a Sb_2_S_3_ shell of approximately 10 nm, in which the presence of a senarmontite Sb_2_O_3_ phase was clearly revealed [55]. No optimization for the Sb_2_S_3_ shell purity was shown, although it is well-known to significantly influence the photovoltaic performance. The oxidation of the bulk of the Sb_2_S_3_ absorber layer has been proven to be detrimental to the photovoltaic performance of solar cells in both planar and nanostructured configurations, because Sb_2_O_3_ creates deep traps that cause the recombination of generated charge carriers [38,62]. Surface oxidation is by some accounts a valid technique for passivating the surface of the Sb_2_S_3_ absorber to boost the PCE [39], although the record PCE of 7.5% was reached with a post-sulfurized absorber [38]. More recently, significant efforts have been achieved on the development of a Sb_2_S_3_ shell by standard successive ionic layer adsorption and reaction (SILAR) [56] and spin-coating-assisted SILAR [58,59,60] techniques. The fabrication of ZnO/TiO_2_/Sb_2_S_3_ core–shell NW heterostructures with a higher purity of the Sb_2_S_3_ shell was shown by standard SILAR process through the formation of three-dimensional clusters [56]. The use of spin-coating-assisted SILAR to form a Cu-doped Sb_2_S_3_ shell modulating the bandgap alignment with ZnO NWs further resulted in the fabrication of sensitized-solar cells with a PCE of 3.14% [58]. The verticality and length of ZnO NWs were also found to strongly affect the photovoltaic performances of Sb_2_S_3_-sensitized solar cells, resulting in a typical PCE of around 2% after optimization [59,60]. The SILAR-related techniques have a high potential in the field of Sb_2_S_3_-sensitized solar cells, but typically lead to the formation of three-dimensional clusters or dots [56,58,59,60], which, in contrast to the CSP technique, results in the formation of a continuous thin shell for Sb_2_S_3_-based ETA solar cells [55]. Interestingly, the recent development of the CSP of Sb_2_S_3_ thin films by using a two-step process has further improved the deposit continuity as compared to the one-step process, while drastically increasing its purity by suppressing the formation of the senarmontite Sb_2_O_3_ phase, in turn increasing the PCE to 5.5% [63]. Actually, the highest efficiencies in Sb_2_S_3_-sensitized solar cells when grown by chemical deposition techniques have, to date, been only achieved by using an amorphous Sb_2_S_3_ phase as the first step.

In this study, the ZnO/TiO_2_/Sb_2_S_3_ core–shell NW heterostructures grown by low-cost chemical deposition techniques, along with P3HT as the HTM and Au as the top electrode, are developed for ETA solar cells. We optimize the thickness of the Sb_2_S_3_ shell, whereas the ZnO NW/TiO_2_ stack below the absorber is intentionally kept constant to decouple the physical phenomena at work. We further modify the growth conditions of the Sb_2_S_3_ shell by revising the sulfur content in the CSP solution to suppress the oxidation of the bulk of the Sb_2_S_3_ shell during deposition. The effect of the Sb_2_S_3_ shell thickness on the structural and optical properties of ZnO/TiO_2_/Sb_2_S_3_ core–shell NW heterostructures, as well as on the performances of the resulting ETA solar cells, is investigated in detail by field-emission scanning electron microscopy (FESEM) imaging, in-plane X-ray diffraction (XRD), Raman and cathodoluminescence spectroscopy, UV–visible absorption and current density (*J*)–voltage (*V*) measurements under dark and air mass (AM) 1.5 G illumination conditions. Our investigation aims at identifying and clarifying the materials and technological issues limiting the performances of ZnO NW-based ETA solar cells, while proposing innovative solutions.

## 2. Materials and Methods

The ZnO/TiO_2_/Sb_2_S_3_ core–shell NW heterostructures were grown by low-cost and easily scalable techniques on indium tin oxide (ITO)–glass substrates. The 150 nm–thick ITO layer (Delta Technologies, Loveland, CO, USA) had a sheet resistance in the range of 5–15 Ω/sq. and an optical transmittance larger than 85%. Its sheet resistance after a thermal treatment at a temperature higher than 400 °C was increased to a typical value in the range of 40–80 Ω/sq. ZnO NW arrays were grown by a two-step wet chemical route. First, the polycrystalline ZnO seed layers were deposited by sol–gel process. An equimolar solution of zinc acetate dihydrate (Zn(CH_3_OOH)_2_·2H_2_O, Sigma-Aldrich, St. Louis, MO, USA) and monoethanolamine (MEA, Sigma-Aldrich, St. Louis, MO, USA) was mixed in pure ethanol. The sol was stirred for 12 h at 60 °C and then for 12 h at room temperature to complete the dissolution of Zn(CH_3_OOH)_2_·2H_2_O. The xerogel film was formed by dipping the ITO–glass substrates into the sol and by withdrawing them at the speed of 3.3 mm/s in ambient atmosphere with a relative humidity below 15%. The xerogel film was put for 10 min on a hot plate kept at 300 °C to evaporate the organic compounds, and then annealed for 1 h in an oven kept at 500 °C to crystallize the ZnO seed layer. Second, the ZnO-seed-layer-coated ITO–glass substrates were placed face down in a sealed reactor to form ZnO NWs by CBD. A 30 mM equimolar solution of zinc nitrate hexahydrate (Zn(NO_3_)_2_·6H_2_O, Sigma-Aldrich, St. Louis, MO, USA) and hexamethylenetetramine (HMTA, Sigma-Aldrich, St. Louis, MO, USA) was prepared in deionized water. The sealed reactor was kept at 90 °C for 3 h in a regular oven. Subsequently, ZnO NW arrays were covered by ALD with an amorphous 10 nm–thick TiO_x_ layer grown at 200 °C from *tetrakis*-dimethylamino titanium (TDMAT) and H_2_O in a F200 Fiji reactor from Cambridge Nanotech (Cambridge, MA, USA). A post-deposition thermal treatment in air was performed for 3 h in a regular oven kept at 300 °C to crystallize the anatase TiO_2_ shell. Eventually, after purifying the sample surface under UV–ozone treatment for 30 min at room temperature in ambient air, an amorphous Sb_2_S_3_ shell was deposited by ultrasonic CSP in air at 210 °C from a solution of 60 mM antimony chloride (SbCl_3_, Sigma-Aldrich, St. Louis, MO, USA) and 180 mM thiourea (Alfa Aesar, Ward Hill, MA, USA) dissolved in 99.8 vol% methanol (Honeywell, Charlotte, NC, USA) [63]. The deposition time was varied in cycles, where one cycle lasts 20 s, as described previously in Reference [63]. Afterward, Sb_2_S_3_ was crystallized in a tubular furnace in flowing 99.999% N_2_ at 300 °C for 5 min. To deposit the HTM layer for the ETA solar cell, the samples were immersed in a solution of 2.0 wt% of regioregular poly(3-hexylthiophene-2,5-diyl) (P3HT, Carbosynth, Newbury, UK), dissolved in chlorobenzene (Sigma-Aldrich, St. Louis, MO, USA), and were ultrasonicated for 15 min. Thereafter, the samples were withdrawn from the solution and dried in air at 50 °C for 10 min, followed by a thermal treatment in vacuum (5.3·10^−4^ Pa) at 170 °C for 5 min. The ETA solar cells made of ZnO NW heterostructures were completed by thermally evaporating a layer of 99.999% Au through a mask to form an array of contacts, each 1.7 mm^2^ in area.

FESEM images in top-view and cross-sectional view configurations of the incomplete structure were collected with a ZEISS GeminiSEM 300 instrument (Carl Zeiss, Oberkochen, Germany) operating at an accelerating voltage of 3 kV. FESEM images of the complete structure were collected with a ZEISS HR Ultra 55 instrument (Carl Zeiss, Oberkochen, Germany) operating at an accelerating voltage of 4 kV, and the FESEM–EDX data were therein recorded at an accelerating voltage of 7 kV, using a Bruker ESPRIT 1.8 EDX detector (Bruker, Billerica, MA, USA). In-plane XRD patterns were recorded with a RIGAKU Smartlab diffractometer (Rigaku, Tokyo, Japan) equipped with a 9 kW rotating anode, using the K_α_(Cu) radiation and operating at 45 kV and 200 mA. The in-plane configuration was run on a 5 circle-goniometer that was specifically designed for this type of acquisitions. The X-ray beam was about 2 mm/0.05 nm parallel/perpendicular to the sample surface, respectively. The samples were placed in a horizontal position on a double tilt stage during the acquisition. Moreover, 2Theta-Chi/Phi XRD measurements were performed in the range of 20° to 60°, with a step of 0.04° and a speed of 1.0°/min, while setting the Omega incidence to 0.5°. The bixbyite In_2_O_3_, wurtzite ZnO, anatase TiO_2_, and stibnite Sb_2_S_3_ phases were identified by using the 00-006-0416, 00-036-1451, 00-021-1272, and 00-042-1393 files of the International Center for Diffraction Data (ICDD), respectively. Raman spectra were recorded with a HORIBA/JOBIN YVON Labram spectrometer (Jobin Yvon, Palaiseau, France) equipped with a liquid-nitrogen-cooled CCD detector. The 632.8 nm excitation line of a Ne/He laser was used with a power on the sample surface close to 10 µW. The laser beam was focused to a spot size of 1 μm^2^, using a 100× objective, leading to a power density of 10 µW/µm^2^. The spectra were calibrated in wavenumber at room temperature by using a silicon reference sample where the theoretical position of the silicon Raman line was set to 520.7 cm^−1^. The acquisition time for Raman scattering measurements was 600 s. Then 5 K cathodoluminescence measurements were performed on ZnO/TiO_2_/Sb_2_S_3_ core–shell NW heterostructures with an FEI Inspect F50 FESEM instrument (FEI, Hillsboro, OR, USA) equipped with a liquid-helium-cooled stage. The cathodoluminescence signal was collected through a parabolic mirror and analyzed with a 550 mm focal length monochromator equipped with 600 grooves/mm diffraction grating. Cathodoluminescence spectra were recorded with a thermoelectric-cooled silicon CCD detector over a 3 × 3 µm^2^ area, using a low acceleration voltage of 10 kV and a small spot size (i.e., less than 10 nm), along with an acquisition time of 30 s. Optical total transmittance and total reflectance were measured by using a JASCO V-670 UV–visible–NIR spectrophotometer (Jasco Applied Sciences, Halifax, NS, Canada) equipped with a 40 mm integrating sphere. ETA solar cell *J*–*V* curves under dark and AM 1.5 G illumination conditions were measured by using a factory-calibrated solar simulator (Xe light source, Newport Oriel Sol3A class AAA, Newport, Irvine, CA, USA) and a shadow mask with an aperture larger than the cell area defined by the back contact. The intensity of the solar simulator was calibrated to 100 mW/cm^2^ at AM 1.5 G illumination conditions with a reference silicon solar cell. External quantum efficiency (EQE) spectra were measured at room temperature, using a light source (Newport 300 W Xenon lamp, 69911 equipped with a monochromator Newport Cornerstone 260, Newport, Irvine, CA, USA), a digital lock-in detector (Merlin, Newport, Irvine, CA, USA), and a factory-calibrated silicon reference detector. EQE-integrated *J*_SC_ was calculated in AM 1.5 G illumination conditions with the online tool Open Photovoltaics Analysis Platform (http://web.archive.org/web/20191216042337if_/http://opvap.com/eqe.php, accessed on 15 November 2021).

## 3. Results and Discussion

### 3.1. Structural and Optical Properties

**Figure 1:**
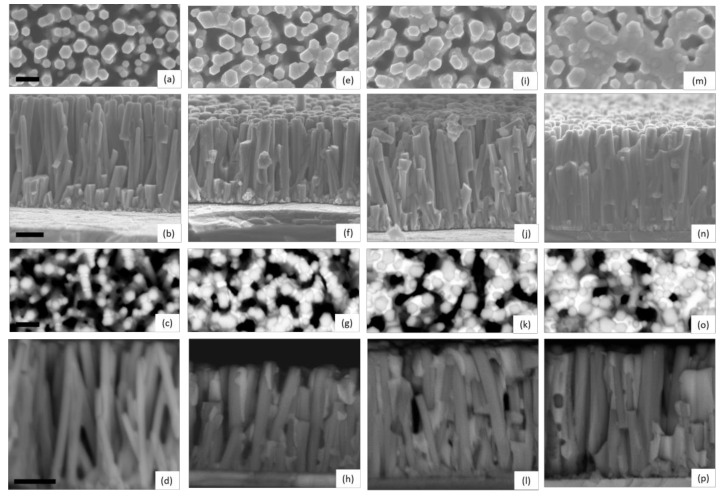
Top-view and cross-sectional view FESEM images of ZnO/TiO_2_ core–shell NW heterostructures covered with a Sb_2_S_3_ shell grown by CSP, using (**a**–**d**) 30, (**e**–**h**) 50, (**i**–**l**) 70, and (**m**–**p**) 90 cycles. FESEM images were collected by using the detectors of secondary electrons (**a**,**b**,**e**,**f**,**i**,**j**,**m**,**n**) and backscattered electrons (**c**,**d**,**g**,**h**,**k**,**l**,**o**,**p**), respectively. The scale bars denote 200 and 300 nm for the top-view and cross-sectional view FESEM images, respectively.

The structural morphology of ZnO/TiO_2_ core–shell NW heterostructures covered with a Sb_2_S_3_ shell grown by CSP, using 30, 50, 70, and 90 cycles, is presented in Figure 1 through FESEM images.

Vertically aligned ZnO NWs are homoepitaxially grown on the grains with polar orientations composing the *c*-axis oriented ZnO seed layer [64,65]. They exhibit a mean length and diameter of 998 ± 115 nm and 92 ± 21 nm, respectively, when using an equimolar concentration of 30 mM [66]. The TiO_2_ shell has a mean thickness of 8.5 nm on the sidewalls of ZnO NWs and of around 10 nm on their top *c*-face, and exhibits a pure anatase phase as determined by HRTEM imaging in Reference [61], which is desirable to get a type II band alignment with ZnO NWs. The ZnO/TiO_2_ core–shell NW heterostructures are, on average, separated from each other by a distance of about 100 nm, forming a gap. A typical top-view FESEM image of ZnO/TiO_2_ core–shell NW heterostructures is shown in Supplementary Materials Figure S1. These heterostructures are well covered by the Sb_2_S_3_ shell from bottom to top, regardless of the cycle number, as seen in Supplementary Materials Figure S2. Overall, the thickness of the Sb_2_S_3_ shell increases as the cycle number is increased. When using 30 cycles, the Sb_2_S_3_ shell appears to be very thin, as indicated by the hexagonal section of ZnO/TiO_2_/Sb_2_S_3_ NWs and by the absence of any connections between them in Figure 1a–d. In contrast, in the cross-sectional view of ZnO/TiO_2_/Sb_2_S_3_ NWs, contours are more rounded when the cycle number is increased to 50 as seen in Figure 1e–h, revealing the thickening of the Sb_2_S_3_ shell through the development of the thin, conformal layer. Moreover, a couple of connections start to be established at that cycle number between closely spaced ZnO/TiO_2_ NWs. The connections between the ZnO/TiO_2_ NWs are even more pronounced at the cycle number of 70, as seen in Figure 1i–l. The gaps between ZnO/TiO_2_ NWs are almost filled by the Sb_2_S_3_ shell at the cycle number of 90, as seen in Figure 1m–p, switching from the core–shell configuration typically used in ETA solar cells to the fully impregnated configuration usually employed in bulk-heterojunction quantum dot solar cells and organic/hybrid solar cells [3]. From the present FESEM data, it is inferred that the thickness of the Sb_2_S_3_ shell varies, on average, from a couple of nanometers to several tens of nanometers as the cycle number is increased from 30 to 90.

**Figure 2:**
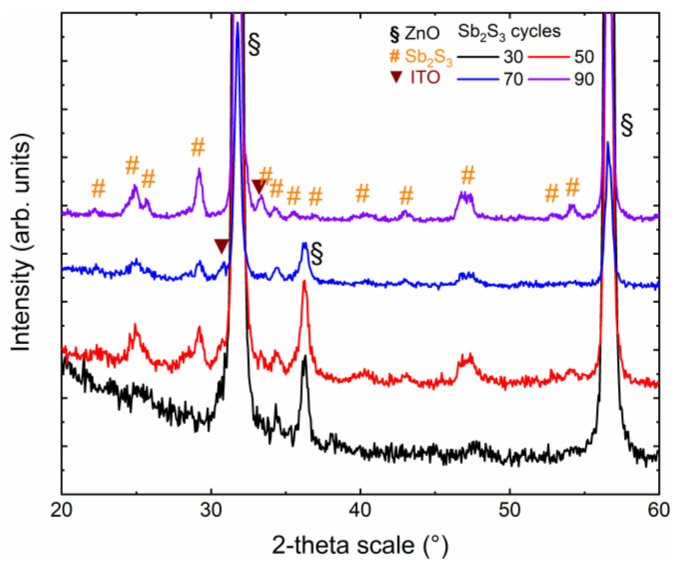
In-plane XRD patterns of ZnO/TiO_2_ core–shell NW heterostructures covered with a Sb_2_S_3_ shell grown by CSP, using 30, 50, 70, and 90 cycles.

The crystallinity and purity of the Sb_2_S_3_ shell, as well as its dependence on the cycle number, were assessed by XRD and Raman spectroscopy. The in-plane XRD patterns of ZnO/TiO_2_ core–shell NW heterostructures covered with a Sb_2_S_3_ shell grown by CSP, using 30, 50, 70 and 90 cycles, are presented in Figure 2.

The in-plane configuration is well-designed to measure the diffracting planes of the Sb_2_S_3_ shell that are perpendicular to the sample surface, namely along the growth axis on the sidewalls of ZnO NWs. The XRD patterns are dominated by the $\left(10\bar{1}0\right)$ and $\left(11\bar{2}0\right)$ diffraction peaks located at 31.7° and 56.6°, respectively, corresponding to the wurtzite phase of vertically aligned ZnO NWs exhibiting the six-fold sidewalls with the nonpolar *m*-planes. The weaker $\left(10\bar{1}1\right)$ diffraction peak at 36.3° indicates that some of the ZnO NWs are slightly tilted with respect to the normal to the sample surface, following their nucleation over some grains with the same semipolar orientation composing the ZnO seed layer. The even weaker diffraction peak at 30.6° is attributed to the (222) planes of the bixbyite phase of the ITO layer. Its weak intensity originates from the in-plane configuration used, in which the X-ray beam was centered on the ZnO/TiO_2_/Sb_2_S_3_ core–shell NW heterostructures. More importantly, the remaining diffraction peaks are all attributed to the stibnite phase of the Sb_2_S_3_ shell. The XRD pattern of the ZnO/TiO_2_ core–shell NW heterostructures covered by the Sb_2_S_3_ shell grown with 30 cycles does not show any intense peaks. The weak diffraction peaks at 25.0°, 34.3°, and around 47.5° correspond to the (130)/(310), (131)/(311), and (002)/(151)/(511) planes. The very thin Sb_2_S_3_ shell grown with 30 cycles was, thus, partially crystallized. As the cycle number is increased to 90, the XRD patterns of the ZnO/TiO_2_ core–shell NW heterostructures covered by the Sb_2_S_3_ shell exhibit a larger number of diffraction peaks with a higher intensity coming from the stibnite phase. Regardless of the cycle number beyond 30, the diffraction peaks at 24.93°, 29.20°, and around 46.80° corresponding to the (130)/(310), (121)/211), and (530)/(002)/(151)/(511) planes, respectively, dominate. A list of all of the diffraction peaks, along with their exact position and nature, is given in Supplementary Materials Table S1. Correlatively, the intensity of the ITO- and ZnO-related diffraction peaks decreases. Interestingly, no sign of the presence of the senarmontite Sb_2_O_3_ phase occurs in the XRD patterns, indicating the absence of this minor phase that was typically detected when Sb_2_S_3_ has been grown by CBD [67]. Additionally, no significant shifts in the position of the diffraction peaks of the ZnO NWs and of the TiO_2_ and Sb_2_S_3_ shells are detected, indicating that the ZnO/TiO_2_/Sb_2_S_3_ core–shell NW heterostructures are fully relaxed. The local epitaxy between the ZnO NWs and TiO_2_ shell grown by ALD is plastically accommodated, such that the epitaxial strain is totally relieved [61]. The Sb_2_S_3_ shell grown by CSP follows a Volmer–Weber growth mode, where the intrinsic stress can be relieved by different processes occurring at grain boundaries [68].

**Figure 3:**
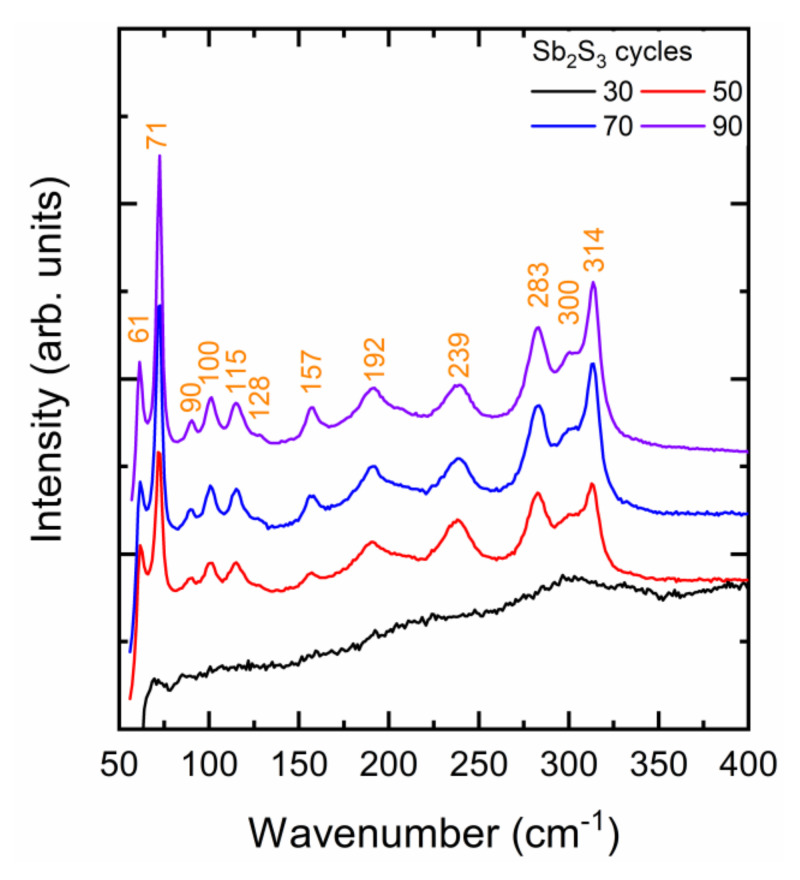
Raman spectra of ZnO/TiO_2_ core–shell NW heterostructures covered with a Sb_2_S_3_ shell grown by CSP using 30, 50, 70, and 90 cycles.

The Raman spectra of ZnO/TiO_2_ core–shell NW heterostructures covered with a Sb_2_S_3_ shell grown by CSP using 30, 50, 70, and 90 cycles are presented in Figure 3. A very small laser power density of 10 µW/µm^2^ was used to avoid any photo-induced degradation of the Sb_2_S_3_ phase, as reported in Reference [69]. The stibnite Sb_2_S_3_ phase belongs to the *Pbnm* (centrosymmetric) space group. According to the factor group analysis at the Γ point, the orthorhombic stibnite structure exhibiting 20 atoms per Sb_2_S_3_ primitive cell has 30 active Raman modes: Γ_Raman_ = 10 A_g_ + 5 B_1g_ + 10 B_2g_ + 5 B_3g_ [70,71,72].

The Raman spectrum of the ZnO/TiO_2_ core–shell NW heterostructures covered by the Sb_2_S_3_ shell grown with 30 cycles does not show any intense narrow lines. Rather, a broad Raman band centered at around 300 cm^−1^ is observed and is typical of the amorphous Sb_2_S_3_ phase [67,73]. This confirms that the very thin Sb_2_S_3_ shell grown with 30 cycles is mainly amorphous and has only been partially crystallized. Any role of chlorine residues in that process due to the lower deposition temperature is ruled out by the fact that chlorine is known to improve the crystallinity of Sb_2_S_3_ thin films [74]. Instead, more and more energy (i.e., higher annealing temperature) is required to crystallize the Sb_2_S_3_ shell at nanoscale dimensions, below a typical thickness of 10 nm. The need for crystallizing layers with a thickness of less than 10 nm at a substantially higher annealing temperature than the same layers with a thickness of several tens of nanometers has been related to an increase in the activation energy for the crystallization process in oxides and phase change materials [75,76,77]. For extremely thin layers, the surface-over-volume ratio is even so high that the surface energy predominantly contributes to the total free energy hampering the amorphous-to-crystalline state transformation below a critical thickness [77]. As the cycle number is increased to 90, the Raman spectra of the ZnO/TiO_2_ core–shell NW heterostructures covered by the Sb_2_S_3_ shell show intense narrow lines originating from the stibnite phase. Regardless of the cycle number beyond 30, the Raman lines at 61 cm^−1^ (B_1g_/B_3g_), 71 cm^−1^ (A_g_), 283 cm^−1^ (A_g_), and 314 cm^−1^ (B_2g_) dominate [70,71,72,78]. A list of all of the Raman lines, along with their exact position and nature, is given in Supplementary Materials Table S2. The Raman line intensity generally increases as the cycle number is increased. This confirms that the Sb_2_S_3_ shell was well-crystallized and that its thickness increased as the cycle number does, which is in agreement with the FESEM and in-plane XRD measurements. Additionally, it should be noted that two Raman lines at 90 and 115 cm^−1^ are detected when the cycle number lies in the range of 50–90. The assignment of these Raman lines is still under debate. On the one hand, they can be attributed to the senarmontite Sb_2_O_3_ phase through the B_2_ and E modes, respectively [79]. The formation of that senarmontite phase takes place when the Sb_2_S_3_ phase is in contact with air, usually passivating the surface layer and thus being beneficial for the heterojunction interface. On the other hand, it was reported in Reference [72], from density-functional theory calculations, that two B_2g_ modes originating from the stibnite phase lie in the same range of wavenumber. In the present case, on the basis of the in-plane XRD patterns and in the absence of the usually dominant Raman lines at around 190 and 255 cm^−1^ belonging to the senarmontite Sb_2_O_3_ phase [67,73], it is deduced that the two Raman lines at 90 and 115 cm^−1^ are attributed to the B_2g_ modes of the Sb_2_S_3_ phase. The absence of the Sb_2_O_3_ phase can be explained by the likely oxidation suppression, because the deposition temperature of the Sb_2_S_3_ shell was decreased from 220 °C in our previous study [55] to 210 °C in this study. Furthermore, the concentration of the Sb and S precursors as SbCl_3_ and thiourea in the CSP process was doubled. Increasing the concentration of the sulfur source in the spray solution is known to suppress the oxidation of metal sulfide layers during the deposition [80].

**Figure 4:**
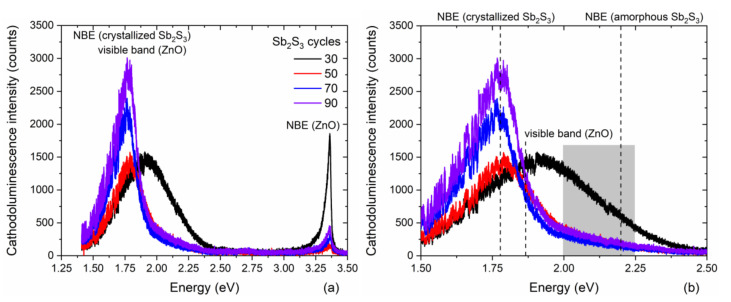
(**a**) 5 K cathodoluminescence spectra collected on an ensemble of single ZnO/TiO_2_ core–shell NW heterostructures covered with a Sb_2_S_3_ shell grown by CSP using 30, 50, 70, and 90 cycles. A fixed surface area of 3 × 3 µm^2^ was chosen for the acquisition. (**b**) Zoom-in in the area of interest corresponding to the visible energy range from 1.5 to 2.5 eV.

The 5 K cathodoluminescence spectra of ZnO/TiO_2_ core–shell NW heterostructures covered with a Sb_2_S_3_ shell grown by CSP using 30, 50, 70, and 90 cycles are presented in Figure 4 following an analysis over a fixed surface area of 3 × 3 µm^2^.

These spectra are composed of three typical emission bands located at 3.36 eV, in the range of 2.00–2.25 eV and at around 1.80 eV, as seen in Figure 4a. The 3.36 eV line corresponds to the near-band edge (NBE) emission of ZnO NWs, which is assigned to donor-bound A exciton transitions involving hydrogen-related defects [81]. From the *I* nomenclature used to label the excitonic transitions [82], the I_4_ and I_5_ lines assigned to substitutional hydrogen on the oxygen lattice site [83] and zinc vacancy/hydrogen defect complexes [84], along with radiative transitions involving interstitial hydrogen in bond centered sites, significantly contribute to the NBE emission of ZnO NWs grown by CBD [81]. The yellow-green emission band at around 2.25 eV is attributed to the (*V*_Zn_-2H) defect complex, while the red-orange emission band at around 1.86 eV is assigned to the (*V*_Zn_-H) defect complex. No green-blue emission band centered at around 2.65 eV and associated with hydrogen-related defects on the surfaces of ZnO NWs occurs, indicating, as expected, that the TiO_2_ shell efficiently passivates the surface defects [61]. Furthermore, it should be noted that the visible emission band lies in the range of 1.50–2.25 eV when the Sb_2_S_3_ shell is grown for 30 cycles, and thus is much broader than the visible emission band lying in the range of 1.50–1.90 eV when the Sb_2_S_3_ shell is grown for 50, 70, and 90 cycles, as seen in Figure 4b. The much lower intensity of the radiative transitions extending beyond 1.90 eV when the Sb_2_S_3_ shell is grown for 30 cycles reveals that the NBE emission of amorphous Sb_2_S_3_ takes place in the yellow-green emission band that is close to its bandgap energy of 2.2 eV [85]. The contribution of the amorphous Sb_2_S_3_ shell to the yellow-green emission band vanishes at a cycle number beyond 30, which is in agreement with in-plane XRD and Raman scattering measurements. Moreover, the NBE emission of the crystallized Sb_2_S_3_ shell occurs in the red emission band that is close to its bandgap energy lying in the range of 1.70–1.80 eV [33,34,85]. As the cycle number is increased from 30 to 90, the ratio of the NBE emission of ZnO NWs over the red-orange emission band significantly decreases. A prominent contribution around 1.78 eV corresponding to the red emission is mainly attributed to the NBE emission of the crystallized Sb_2_S_3_ shell. As the shell thickness is increased with a higher cycle number in the CSP process, the red emission band becomes more and more intense. However, the increase of around 20% in the intensity of the red emission band when the Sb_2_S_3_ shell is grown with a cycle number of 90, as compared to 70, is less than the estimated 30% increase in the thickness of the Sb_2_S_3_ shell. This is likely the sign of a small increase in the density of defects in the bulk of the Sb_2_S_3_ shell when grown with a cycle number of 90.

**Figure 5:**
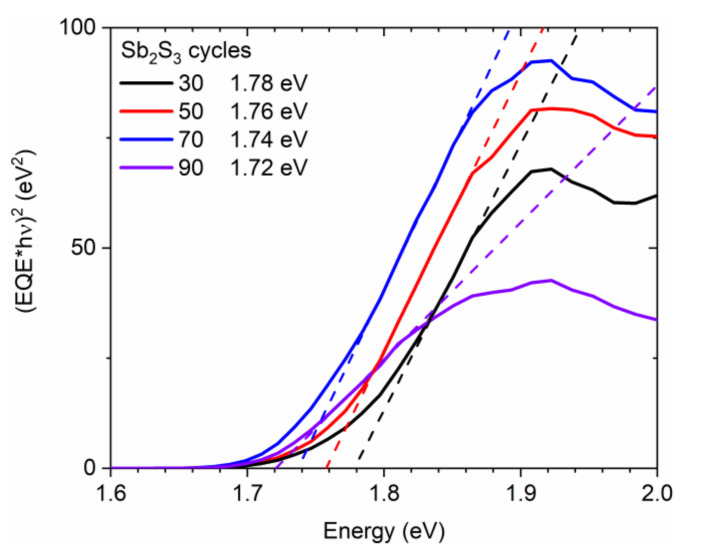
(EQE × hν)^2^ vs. photon energy (solid line) of ZnO/TiO_2_ core–shell NW heterostructures covered with a Sb_2_S_3_ shell grown by CSP, using 30, 50, 70, and 90 cycles. The fitting dashed lines reveal the optical bandgap energy as the intercept of the linear part with the photon energy axis.

Correlatively, the sample color switches from translucent orange to opaque dark brown by increasing the cycle number, which reveals an increase in the absorption of the visible light by the Sb_2_S_3_ shell. The optical bandgap energy of the Sb_2_S_3_ shell was extracted from EQE measurements following the (EQE × hν)^2^ method [86] and is reported in Figure 5. The (EQE × hν)^2^, as a function of photon energy, was plotted, and a linear regression fitting provided the value of the optical bandgap energy. The optical bandgap energy obtained for 30, 50, 70, and 90 cycles is 1.78, 1.76, 1.74, and 1.72 eV, respectively, thus converging to the theoretical value of around 1.7 eV in bulk Sb_2_S_3_ when the shell thickness is increased [33,34,85].

### 3.2. Photovoltaic Performances

**Figure 6:**
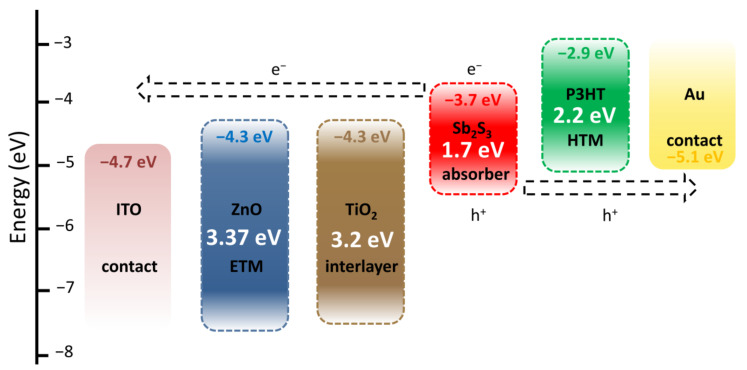
Architecture and corresponding diagram of energy levels of ZnO/TiO_2_/Sb_2_S_3_ core–shell NW heterostructure-based ETA solar cells.

The architecture of ZnO/TiO_2_/Sb_2_S_3_ core–shell NW heterostructure-based ETA solar cells and the corresponding diagram of energy levels are presented in Figure 6. Under AM 1.5 G illumination through the glass substrate, visible photons are absorbed by the Sb_2_S_3_ shell with a bandgap energy of around 1.7 eV to generate electron–hole pairs. The successive type II band alignments are favorable for the separation and collection of electrons and holes to generate the photocurrent as follows: electrons migrate to the TiO_2_ shell and then to the ZnO NWs, acting as the ETM toward their collection with the ITO contact as the topside electrode, while holes migrate to P3HT, acting as the HTM toward their collection with the Au contact as the backside electrode. The TiO_2_ shell acts as a protective, passivating layer [61], further improving the quality of the interface between the ZnO NWs and Sb_2_S_3_ shell. The absence of the TiO_2_ shell very strongly degrades the photovoltaic performances in the present ETA solar cells [55].

**Figure 7:**
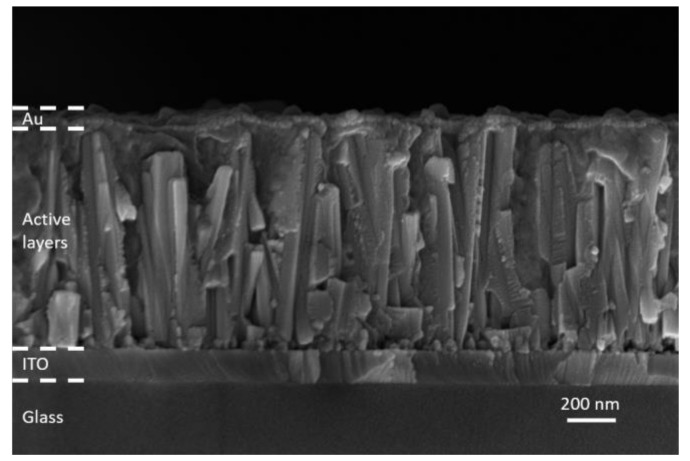
Cross-sectional view FESEM image of the complete ETA solar cell structure of ITO/ZnO/TiO_2_/Sb_2_S_3_/P3HT/Au when using the cycle number of 70.

The ZnO/TiO_2_ core–shell NW heterostructures covered with a Sb_2_S_3_ shell grown by CSP, using 30, 50, 70, and 90 cycles, were filled by P3HT, using immersion and thereafter covered with a thin layer of Au by thermal evaporation to form the complete ETA solar cell structure of ITO/ZnO/TiO_2_/Sb_2_S_3_/P3HT/Au. A cross-sectional view FESEM image of the entire ETA solar cell, as presented in Figure 7, clearly indicates the efficient penetration of the Sb_2_S_3_ shell and P3HT when using the cycle number of 70.

**Figure 8:**
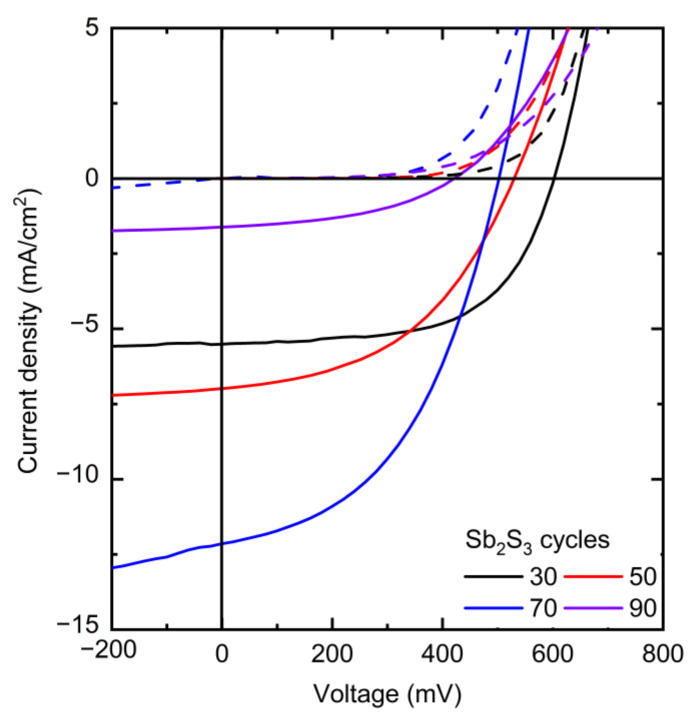
*J*–*V* curves of the best ETA solar cells made of ZnO/TiO_2_ core–shell NW heterostructures covered with a Sb_2_S_3_ shell grown by CSP, using 30, 50, 70, and 90 cycles, collected under dark (dashed lines) and AM 1.5 G illumination (solid lines) conditions.

**Figure 9:**
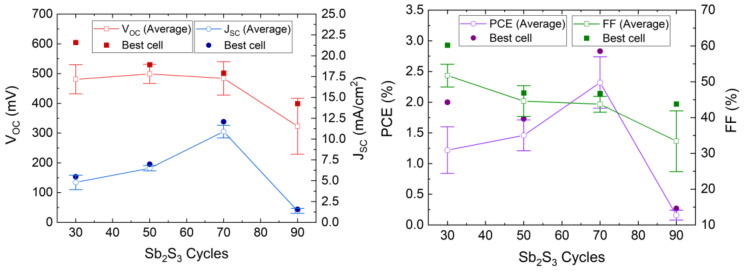
Evolution of the V_OC_, J_SC_, FF, and PCE values of the ETA solar cells made of ZnO/TiO_2_ core–shell NW heterostructures covered with a Sb_2_S_3_ shell grown by CSP as a function of the cycle number. Horizontal bars denote standard deviation.

The *J*–*V* curves collected in dark and AM 1.5 G illumination conditions are presented in Figure 8, and the photovoltaic properties are reported in Table 1 and plotted in Figure 9.

The photovoltaic performances of the ETA solar cells involving the ZnO/TiO_2_/Sb_2_S_3_ core–shell NW heterostructures offer a clear trend of the dependence of its characteristics on the Sb_2_S_3_ shell thickness. V_OC_ is quite high, with a mean value above 400 mV when the Sb_2_S_3_ shell is grown for 30, 50, and 70 cycles. The V_OC_ mean value initially increases from 481 to 499 mV as the cycle number is increased from 30 to 50, and then it decreases continuously to 323 mV as the cycle number reaches a value of 90. The initial increase in the V_OC_ mean value is related to a more continuous and better crystallinity of the Sb_2_S_3_ shell when grown with a cycle number of 50, in turn decreasing the density of defects in its bulk. The further decrease in the V_OC_ mean value likely results from the two following major reasons. First, we expect that the growth of a thicker Sb_2_S_3_ shell over high-aspect-ratio ZnO NWs in an ETA solar cell results in the increase in the density of defects in its bulk, as revealed in the cathodoluminescence spectra. Second, the spatial variance of the thickness of the Sb_2_S_3_ shell is expected to appear as the spacings get narrower, which further leads to the issue of incomplete immersion of P3HT into the gaps between the ZnO NWs in the case of the largest Sb_2_S_3_ thickness at 90 cycles, as supported in Supplementary Materials Figure S3. By performing an FESEM–EDX analysis over a given rectangular area located on the cross-section of the ETA solar cells, it is revealed that the Sb/Zn element ratio continuously increases as the Sb_2_S_3_ shell is thickened and, more importantly, that the S/Sb element ratio gradually decreases toward a value of 1.7 as the cycle number is increased to 90. This indicates that the amount of excess sulfur coming from P3HT at a given thickness of the entire ETA solar cells strongly decreases down to a S/Sb element ratio value close to 1.5, which is expected from the sulfur coming only from the Sb_2_S_3_ shell. As such, the penetration depth of the P3HT is reduced by the thickening of the Sb_2_S_3_ shell, raising the issue of a progressive incomplete immersion of P3HT as the cycle number is increased from 30 to 90. Furthermore, it is well-known from ETA solar cell studies that the uniformity of the thickness of the absorber shell is a critically important performance factor [3,55]. Consequently, the heterojunction quality worsens as the cell conceptually changes by gradually transforming from the ETA cell type to a 3D cell type in which the active component is not a conformal shell anymore, leading to the observed drop in the V_OC_. In contrast, the J_SC_ mean value significantly increases from 4.80 to 10.90 mA/cm^2^ as the cycle number is increased to 70, which can be attributed to an enhancement of the optical absorption that increases the charge carrier generation rate thanks to the increase in the thickness of the Sb_2_S_3_ shell. However, the J_SC_ mean value then falls to 1.38 mA/cm^2^ as the cycle number reaches 90. In particular, as discussed above, at larger cycle numbers to deposit the Sb_2_S_3_ shell, the increased density of defects in its bulk induces more recombination while the ZnO NWs exhibit less gap space to accommodate HTM, with both of them leading to poor charge carrier collection and extraction. In fact, in order to reach the HTM in the ETA solar cells with a thick Sb_2_S_3_ shell and a poor HTM coverage, the holes are expected to move along the Sb_2_S_3_ shell for as much as the length of the core–shell NWs, in excess of 1000 nm, which is five times longer than the diffusion length of charge carriers reported for Sb_2_S_3_ [87]. Recombination thus occurs before the holes reach the HTM.

The calculated fill factor (FF) also decreases continuously from the mean values of 51.7 to 33.4% as the cycle number is increased up to 90; hence, its trend generally follows the trend of the V_OC_. The series resistance (R_s_) mean values are 24.8, 26.8, 15.3 and 127 Ω·cm^2^ for a cycle number of 30, 50, 70, and 90, respectively. This indicates that, at a cycle number of 70, the thickness of the Sb_2_S_3_ shell yields the lowest R_s_, and increasing to a cycle number of 90 immediately causes an eightfold increase in R_s_, as is apparent from the trend in J_SC_. The shunt resistance (R_sh_) mean values are 1394, 681, 343, and 630 Ω·cm^2^ for a cycle number of 30, 50, 70, and 90, respectively. Both R_sh_ and V_OC_ decrease as the cycle number is increased. Thus, the poor penetration of P3HT into the space between core–shell NWs combined with the uneven thickness of the Sb_2_S_3_ coating could possibly cause a reduction in charge carrier transfer from Sb_2_S_3_ to P3HT. Thereby, interface recombination is increased, leading to a reduction in R_sh_. In addition, Sb_2_S_3_ shells interconnecting at closely positioned core–shell NWs could in turn cause shunting (i.e., short-circuits). Furthermore, the reduction of R_sh_ as the cycle number is increased could also be due to the increased density of defects in the bulk of the Sb_2_S_3_ shell. The PCE mean value of the ETA solar cells increases from 1.22 to 2.32% as the cycle number is increased from 30 to 70, and then it decreases drastically to 0.16% as the cycle number is further increased to 90. The evolution of the PCE mean value is thus driven strongly by the evolution of the J_SC_ mean value, along with that of the V_OC_ mean value to a lesser extent. The best ETA solar cell is obtained when the Sb_2_S_3_ shell is grown for 70 cycles, with a maximum PCE of 2.83% (V_OC_ = 502 mV, J_SC_ = 12.08 mA/cm^2^, and FF = 46.7%). The increase in the thickness of the Sb_2_S_3_ shell is favorably related to an improvement of the optical absorption of visible photons generating more charge carriers, but a balance must be found, as its further increase is liable to increase the density of defects in its bulk and lead to the poor penetration depth of the HTM, both of which likely cause the reduced hole collection. As the distance between bare core–shell NWs is constant, as seen in Figure 1, a thicker absorber inevitably leads to a narrower and shallower space between the core–shell NWs for the HTM to occupy. As a result, the penetration depth of the HTM decreases at a larger Sb_2_S_3_ thickness, which is evident in the performance drop above 70 cycles. The optimization of the Sb_2_S_3_ shell thickness thereby results in an increase in the PCE value of over 0.5%, as compared to our first ETA solar cells reported in Reference [55].

**Figure 10:**
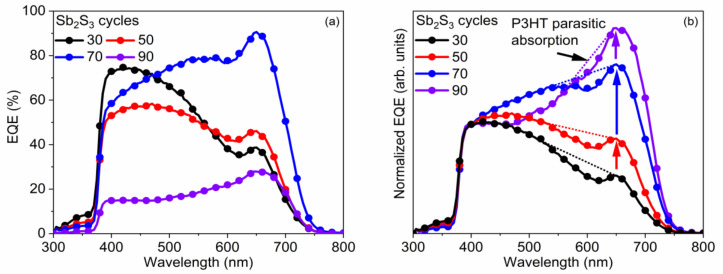
(**a**) EQE vs. wavelength and (**b**) normalized EQE to its value at 400 nm vs. wavelength of the best ETA solar cells made of ZnO/TiO_2_ core–shell NW heterostructures covered with a Sb_2_S_3_ shell grown by CSP, using 30, 50, 70, and 90 cycles. The dotted lines are a guide-to-the-eye, lying roughly between the absorption onset of P3HT at 650 nm on one side, and the absorption maximum of P3HT, at 525 nm, on the other side.

**Figure 11:**
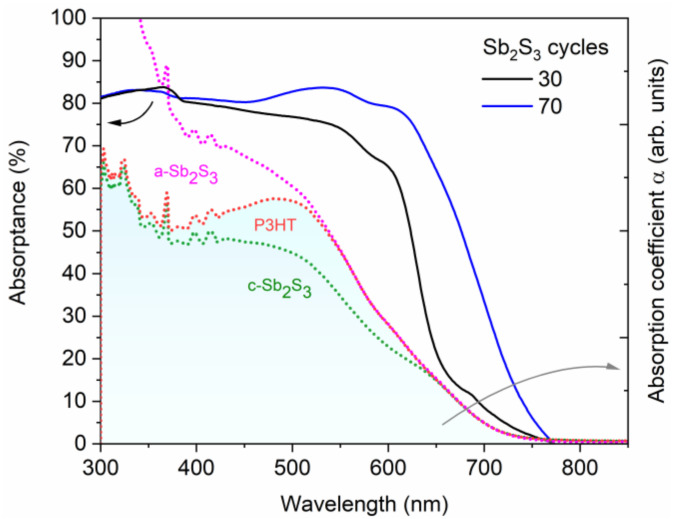
Absorptance (solid lines) of the best ETA solar cells made of ZnO/TiO_2_ core–shell NW heterostructures covered with a Sb_2_S_3_ shell grown by CSP, using 30 and 70 cycles, and topped by P3HT. Absorption coefficient, α (dashed lines), of each layer and of the expected additional phase (a-Sb_2_S_3_) in the ETA cell. The absorption coefficients of P3HT and amorphous Sb_2_S_3_ (a-Sb_2_S_3_) were elevated above crystalline Sb_2_S_3_ (c-Sb_2_S_3_) and P3HT, respectively, to illustrate the cumulative effect of each added layer on the optical density of the ETA cell.

The EQE measurements of the best ETA solar cells made of ZnO/TiO_2_ core–shell NW heterostructures covered with a Sb_2_S_3_ shell grown by CSP, using 30, 50, 70, and 90 cycles, are reported in Figure 10, along with the absorptance and absorption coefficient of each layer in Figure 11. By integrating the EQE spectra over the wavelength, the ideal J_SC_ values of 10.2, 9.7, 15.6, and 4.2 mA/cm^2^ for a cycle number of 30, 50, 70, and 90, respectively, corresponding to the best samples reported in Table 1, were obtained, and, as a trend, they are in relative agreement with the J_SC_ values deduced from the *J*–*V* measurements under AM 1.5 G illumination condition. The J_SC_ value calculated from EQE measurements is overall higher than that calculated from *J*–*V* curves, probably due to fundamental differences in illumination intensities for both techniques. There exists a clear positive offset in favor of the values calculated from the EQE data, which were collected by using monochromatic low-intensity light scans. At low illumination intensities, the PCE value (and, thus, the J_SC_ value when normalized to the light intensity) is expected to be higher, as already demonstrated for a planar analogue of this type of cell [88].

The EQE value at around 650 nm, attributed solely to Sb_2_S_3_, as neither P3HT nor any of the other layers in the core–shell NWs absorb light in this region [89], as further shown in Figure 10, increases from 38.7% to 46.1%, peaking at 90.6% as the Sb_2_S_3_ shell is grown for a cycle number increasing from 30 to 70, and then falls to 28.0% for a cycle number of 90. As the cycle number is increased, the EQE at 380–500 nm steadily decreases, as seen in Figure 10a. EQE could decrease in this region because the holes generated by visible photons with a low penetrating depth (25–50 nm based on the absorption coefficient of Sb_2_S_3_ in this range [88]) have to move through a thicker Sb_2_S_3_ shell to reach the HTM. However, as the EQE in this region is higher at a cycle number of 70 vs. 50, it is more likely that the trend is related to the shift in the position at which photons are absorbed in the stack relative to the ITO and Au contacts. The issue of hole collection and extraction is further amplified when the Sb_2_S_3_ shell is grown for a cycle number larger than 70, due to a non-conformal HTM coating, or even the absence of any HTM coating originating from the decreased gap size as discussed earlier. In contrast, the EQE at 550–720 nm increases as the cycle number is increased, peaking at 70 cycles, thanks to the increased optical absorption in the thicker Sb_2_S_3_ shell, whereas enough space still remains for P3HT to penetrate to the deepest parts of the ETA solar cell. Finally, at 90 cycles, the Sb_2_S_3_ shell is already so thick that recombination dominates owing to the poor P3HT penetration, and the entire EQE spectrum has plummeted in intensity. The EQE normalized to the high-energy-absorption edge in Figure 10b illustrates the proportional increase of optical absorption in the low-energy region as the cycle number is increased. The dotted line shows the perceived EQE loss due to the parasitic absorption in the P3HT, as well-established in earlier reports [43], fitting the shape of its absorption that peaks at around 600 nm. As the cycle number is increased, the EQE loss due to the parasitic absorption seems to decrease and span across a smaller wavelength range. This can be explained by a decreasing amount of light reaching the P3HT, as a thicker Sb_2_S_3_ shell absorbs more incident light at the 550–720 nm wavelength range. Evidently, further gains in J_SC_ and PCE values could be achieved for this type of ETA solar cell by making use of a UV–vis transparent HTM to prevent parasitic absorption.

The recapitulated photovoltaic performances of the nanostructured solar cells involving the heterostructures made of ZnO NWs and Sb_2_S_3_, as a comparison with this work, are presented in Table 2. Most of the reported data involve the core–shell configuration at the expense of the fully impregnated configuration, as defined in Reference [3], owing to its higher potential to benefit from the advantages of integrating ZnO NWs in the architecture. In comparison with our previous results in Reference [55], the J_SC_ value of the best device significantly increased from 7.5 to 12.08 mA/cm^2^, while the V_OC_ value decreased from 656 to 502 mV, at a similar FF value of 47%, resulting in an increase in the PCE from 2.3% to 2.83% (Table 1 and Table 2). The V_OC_ and J_SC_ values thus govern the PCE variation in the present study. As shown in Reference [55], the V_OC_ value primarily stems from the use of the TiO_2_ passivating layer and P3HT HTM, with minor contribution from the added Sb_2_S_3_ shell. In contrast, the J_SC_ value is mainly driven by the Sb_2_S_3_ absorber shell. Moreover, it is known that longer ZnO NWs as the ETM generally result in a proportionally larger J_SC_ value, with a small decrease in the V_OC_ value due to added interfacial recombination [90]. Thus, the increase in the J_SC_ value in the current development, as compared to Reference [55], originates from the use of slightly longer ZnO NWs (998 ± 115 nm vs. 900 nm), as well as the enhanced quality and increased thickness of the Sb_2_S_3_ absorber shell. In contrast, the decrease in the V_OC_ value can partially stem from the small increase in the length of ZnO NWs. It should be noted that the recent development of nanostructured solar cells with liquid electrolyte as the HTM has emerged as an alternative to the ETA solar cells with P3HT as the HTM. The direct comparison shows that the J_SC_ values are so far less in these solar cells, which, however, reveal higher FF values. They further raise the problem of stability with time, as in the case of dye-sensitized solar cells, for which the quality of the interface between the inorganic materials and liquid electrolyte still represents a major issue. In that respect, the introduction of an interlayer, such as ZnS [54] and TiO_2_ [55] between the ZnO NWs and Sb_2_S_3_ shell when grown by chemical deposition techniques, appears as a typical route to further optimize the architecture of these ETA solar cells.

These findings show that the dimensional optimization of all the components in the ZnO/TiO_2_/Sb_2_S_3_ core–shell NW heterostructures is crucial to further improve the photovoltaic performance of the related ETA solar cells, while the issue of the HTM is capital to properly collect the charge carriers, specifically the holes. It is foreseen that, by carefully varying the length and aspect ratio of ZnO NWs, as well as the spacing between them, a larger amount of absorber could be incorporated in the core–shell NW heterostructures in a way that the gaps between the core–shells are not absorber-filled, thus promoting a more uniform HTM coating and preserving the integrity of the ETA concept. Moreover, the application of an HTM, which is more transparent in the visible range, could further increase the J_SC_ value to boost the PCE. To accommodate the thicker Sb_2_S_3_ shell, detrimental interconnections between the core–shell NW individual heterostructures must be eliminated by growing each ZnO NW perpendicular to the substrate and parallel to adjacent ZnO NWs in a more optimized arrangement. On the other end, at the extremely low absorber thicknesses, elevated crystallization temperatures are liable to cause materials and technological issues when the substrate cannot handle the elevated thermal load and should be taken into account when modeling and designing experiments.

## 4. Conclusions

In summary, we investigated the effect of the thickness of the Sb_2_S_3_ shell by varying the cycle number from 30 to 90 during the CSP process on the structural and optical properties of the ZnO/TiO_2_/Sb_2_S_3_ NW heterostructures, along with the photovoltaic performance of ETA solar cells, using P3HT as the HTM. By growing the Sb_2_S_3_ shell at the moderate temperature of 210 °C, using the CSP process, the Sb_2_S_3_ shell was found to be of high purity and free of the unwanted senarmontite Sb_2_O_3_ phase. This represents a strong advantage of the CSP process over the CBD process. The limitations at both the low and high end of Sb_2_S_3_ shell thicknesses from a couple of nanometers to several tens of nanometers were discussed in detail. The low end is limited by challenges in the crystallization of the Sb_2_S_3_ shell that is amorphous at nanoscale dimensions, resulting in the low optical absorption of visible photons. In contrast, the high end is limited by the increased density of defects in the bulk of the Sb_2_S_3_ shell, degrading charge carrier dynamics, and by the incomplete immersion of the P3HT in the structure, resulting in the poor hole collection. The best ETA solar cell with the intermediate optimum thickness of the Sb_2_S_3_ shell shows a J_SC_ of 12.1 mA/cm^2^, a V_OC_ of 502 mV, and a PCE of 2.83%. These findings deepen the knowledge of the advantages and limitations of the architecture of ETA solar cells through materials and technological issues, further emphasizing the intricate nature of any dimensional optimization in the structure as a major challenge to boost their overall photovoltaic performance.

## Figures and Tables

**Table 1:** Photovoltaic properties of the ETA solar cells involving ZnO/TiO_2_ core–shell NW heterostructures covered with a Sb_2_S_3_ shell grown by CSP, using 30, 50, 70, and 90 cycles. The mean values and standard deviations are given in brackets.

Cycle Number	V_OC_ (mV)	J_SC_ (mA/cm^2^)	R_s_ (Ω·cm^2^)	R_sh_ (Ω·cm^2^)	FF (%)	PCE (%)	No. of Cells
30	605 (481 ± 49)	5.48 (4.80 ± 0.87)	16.0 (24.8 ± 5.3)	2784 (1394 ± 789)	60.2 (51.7 ± 3.2)	2.00 (1.22 ± 0.38)	12
50	530 (499 ± 32)	6.97 (6.51 ± 0.32)	22.9 (26.8 ± 3.9)	980 (681 ± 442)	46.9 (44.6 ± 4.3)	1.73 (1.46 ± 0.25)	9
70	502 (484 ± 56)	12.08 (10.90 ± 0.76)	15.2 (15.3 ± 1.5)	221 (343 ± 107)	46.7 (43.7 ± 2.2)	2.83 (2.32 ± 0.42)	10
90	399 (323 ± 94)	1.57 (1.38 ± 0.30)	84.9 (127 ± 38)	818 (630 ± 719)	43.8 (33.4 ± 8.5)	0.27 (0.16 ± 0.08)	16

**Table 2:** Recapitulated photovoltaic properties of the nanostructured solar cells involving the heterostructures made of ZnO NWs and Sb_2_S_3_ in the fully impregnated or core–shell configurations according to the definition used in Reference [3].

Materials	Architecture	Sb_2_S_3_ shell	HTM	V_OC_ (mV)	J_SC_ (mA/cm^2^)	FF (%)	PCE (%)	Reference
ZnO/Sb_2_S_3_	Full impregnation	Thermal evaporation	P3HT	450	16.0	40	2.9	[53]
ZnO/ZnS/Sb_2_S_3_	Core–shell	Chemical conversion	P3HT	440	5.57	54	1.32	[54]
ZnO/TiO_2_/Sb_2_S_3_	Core–shell	CSP	P3HT	656	7.5	47	2.3	[55]
ZnO/Sb_2_S_3_	Core–shell	CBD	Electrolyte	438	1.46	31	0.20	[57]
ZnO/Sb_2_S_3_:Cu	Core–shell	SILAR	Electrolyte	580	9.18	59	3.14	[58]
ZnO/Sb_2_S_3_	Core–shell	SILAR	Electrolyte	586	7.02	59	2.43	[59]
ZnO/Sb_2_S_3_	Core–shell	SILAR	Electrolyte	582	5.91	59	2.04	[60]
ZnO/TiO_2_/Sb_2_S_3_	Core–shell	CSP	P3HT	502	12.08	46.7	2.83	This Work

## Data Availability

The data that support the findings of this study are available from the corresponding authors upon reasonable request.

## Supplementary Materials

The following supporting information can be downloaded at https://www.mdpi.com/article/10.3390/nano12020198/s1. Figure S1: Typical top-view FESEM image of ZnO/TiO_2_ core–shell NW heterostructures without any Sb_2_S_3_ shell, Figure S2: High-magnification cross-sectional view FESEM image of ZnO/TiO_2_ core–shell NW heterostructures covered by a Sb_2_S_3_ shell grown by CSP, using 70 cycles, Figure S3: (a) Cross-sectional view FESEM image of the complete ETA solar cell structure of ITO/ZnO/TiO_2_/Sb_2_S_3_/P3HT/Au when using the cycle number of 70, (b) FESEM-EDX spectra collected on the yellow rectangular area as denoted in (a), (c) Element ratio as a function of the cycle number ranging from 30 to 90, Table S1: In-plane XRD data recapitulating the nature and position of the diffraction peaks in this work, as compared to the 00-042-1393 ICDD file, Table S2: Raman scattering data recapitulating the nature and position of the Raman lines in this work.
