# Supplementary material for: Optimization of the Sb2S3 Shell Thickness in ZnO Nanowire-Based Extremely Thin Absorber Solar Cells
Source: Nanomaterials (Basel). 2022 Jan 7;12(2):198. doi: 10.3390/nano12020198 (PMC8777826; doi:10.3390/nano12020198)
Supplement: Supplementary file 1 [file nanomaterials-12-00198-s001.zip › nanomaterials-1524370-supplementary.pdf]

nanomaterials

MDPI

Supplementary Materials

Optimization of the Sb_2S_3 Shell Thickness in ZnO Nanowire-Based Extremely Thin Absorber Solar Cells

Guislain Hector [1], Jako S. Eensalu [2], Atanas Katerski [2], Hervé Roussel [1], Odette Chaix-Pluchery [1], Estelle Appert [1], Fabrice Donatini [3], Ilona Oja Acik [2], Erki Kärber [2,*] and Vincent Consonni [1,*]

1 Université Grenoble Alpes, CNRS, Grenoble INP, LMGP, F-38000 Grenoble, France; guislain.hector@grenoble-inp.fr (G.H.); herve.roussel@grenoble-inp.fr (H.R.); odette.chaix@grenoble-inp.fr (O.C.-P.); estelle.appert@grenoble-inp.fr (E.A.)
2 Laboratory of Thin Film Chemical Technologies, Department of Materials and Environmental Technology, School of Engineering, Tallinn University of Technology, Ehitajate tee 5, 19086 Tallinn, Estonia; jako.eensalu@taltech.ee (J.S.E.); atanas.katerski@taltech.ee (A.K.); ilona.oja@taltech.ee (I.O.A.)
3 Université Grenoble Alpes, CNRS, Grenoble INP, Institut NEEL, F-38000 Grenoble, France; fabrice.donatini@neel.cnrs.fr
* Correspondence: erki.karber@taltech.ee (E.K.); vincent.consonni@grenoble-inp.fr (V.C.)

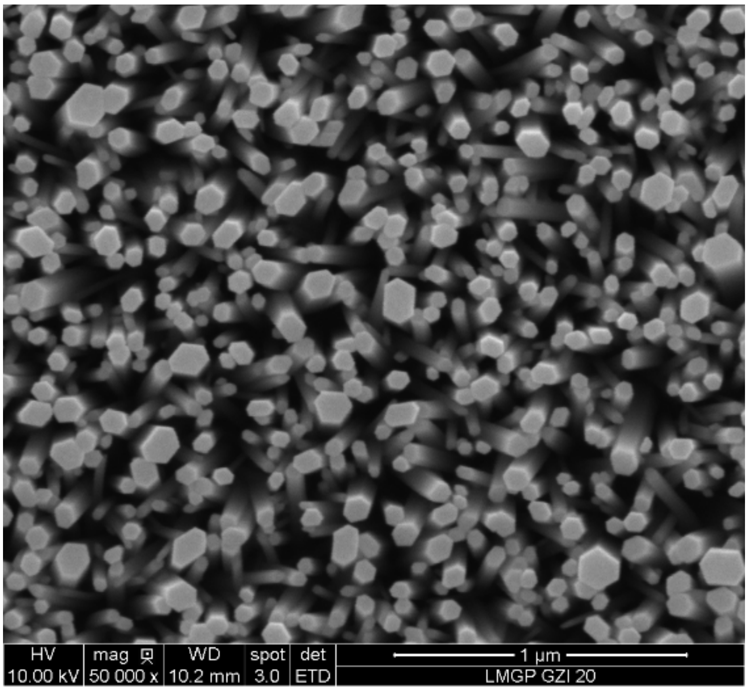

Figure S1. Typical top-view FESEM image of ZnO/TiO_2 core-shell NW heterostructures without any Sb_2S_3 shell.

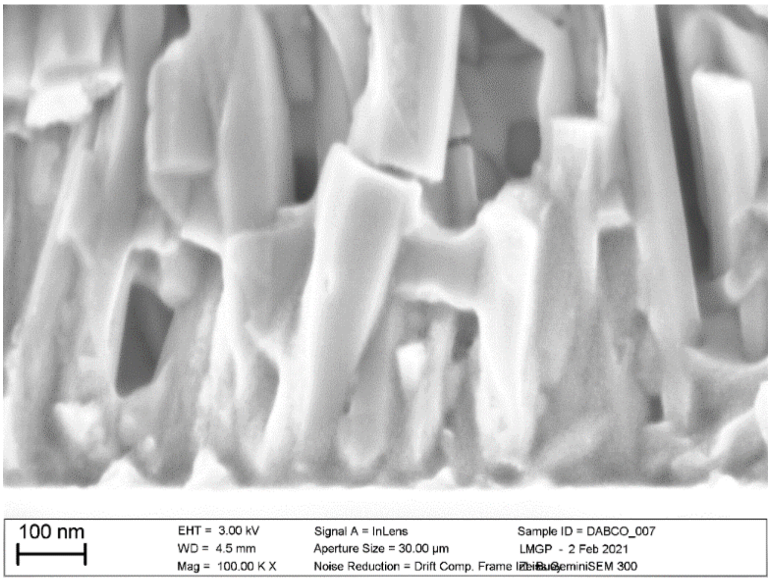

Figure S2. High magnification cross-sectional view FESEM image of ZnO/TiO_2 core-shell NW heterostructures covered by a Sb_2S_3 shell grown by CSP using 70 cycles.

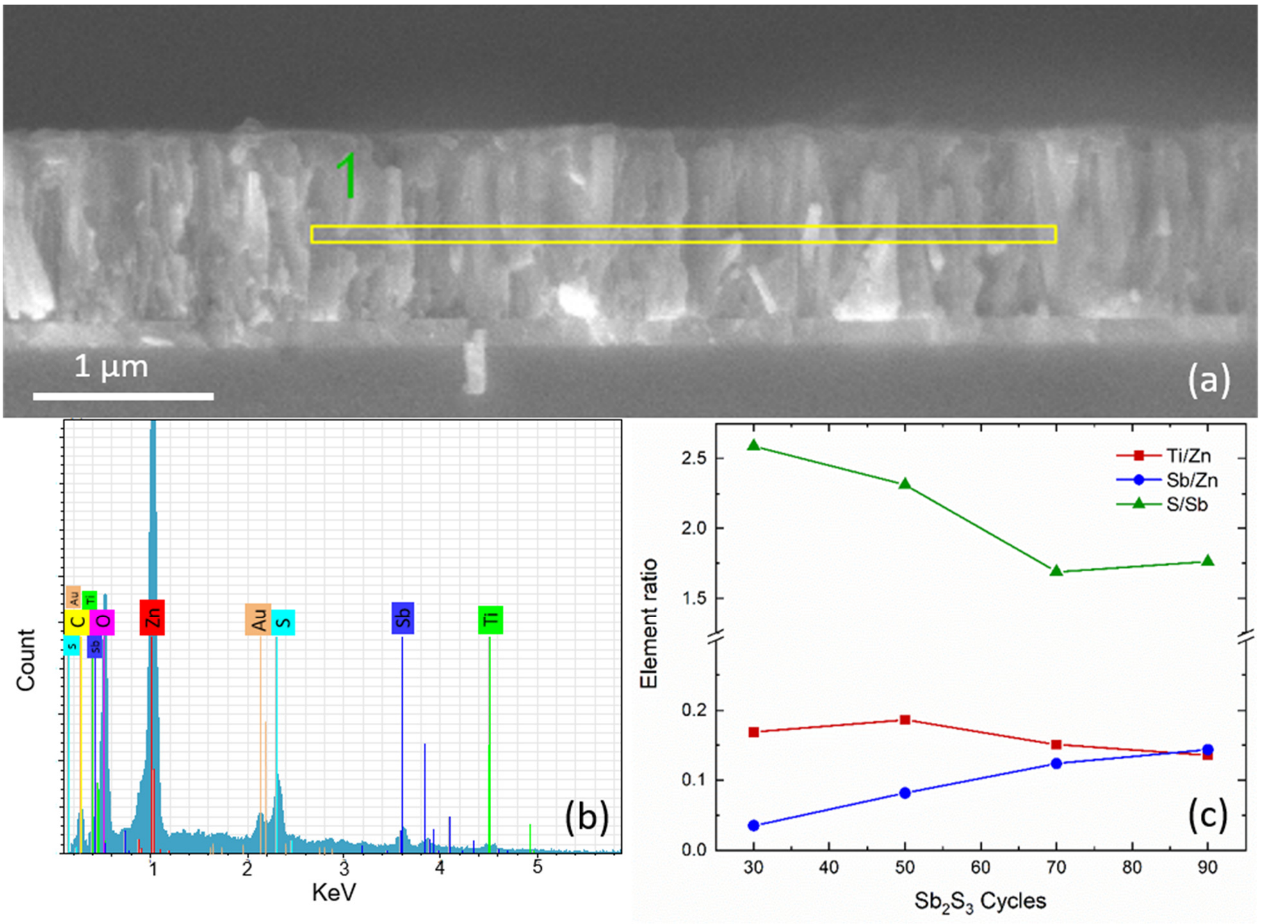

Figure S3. (**a**) Cross-sectional view FESEM image of the complete ETA solar cell structure of ITO/ZnO/TiO_2/Sb_2S_3/P3HT/Au when using the cycle number of 70, (**b**) FESEM-EDX spectra collected on the yellow rectangular area as denoted in (**a**), (**c**) Element ratio as a function of the cycle number ranging from 30 to 90.

Table S1. In-plane XRD data recapitulating the nature and position of the diffraction peaks in this work as compared to the 00-042-1393 ICDD file.

(hkl)	2-Theta (°) This Work	2-Theta (°) 00-042-1393 ICDD file
(220)	22.20	22.28
(130) – (310)	24.93	24.89 – 25.01
(111)	25.65	25.73
(121) – (211)	29.20	29.25
(301)	33.38	33.39
(131) – (311)	34.19	34.24 – 34.34
(240) – (420)	35.54	35.52 – 35.64
(231)	36.76	36.99
(340) – (430) – (141) – (411) – (510)	40.17	39.91 – 40.00 – 40.38 – 40.57 – 40.89
(250) – (520)	42.98	43.04 – 43.30
(530) – (002) / (151) – (511)	46.80	47.04 – 47.30 – 47.54
(531)	52.84	53.04
(061) – (132) / (312) – (360)	54.00	54.13 – 54.20 – 54.41 – 54.65
(630)	54.62	54.65

Table S2. Raman scattering data recapitulating the nature and position of the Raman lines in this work as compared to Ref. [1].

Wavenumber (cm^{-1}) This Work	Wavenumber (cm^{-1}) Ref. [1]	Vibrational modes
61	65.7 / 65.8	B_{1g} / B_{3g}
71	74.7	A_g
90	92.9	B_{2g}
100	100	A_g
115	112	B_{2g}
128	122 / 128	B_{2g} / A_g
157	159 / 159	B_{2g} / A_g
192	190	A_g
239	225 / 227	B_{1g} / B_{3g}
283	280	A_g
300	291 / 293	B_{2g} / A_g
314	294	B_{2g}

References

1. Ibanez, J.; Sans, J.A.; Popescu, C.; Lopez-Vidrier, J.; Elvira-Betanzos, J.J.; Cuenca-Gotor, V.P.; Gomis, O.; Manjon, F.J.; Rodriguez-Hernandez, P.; Munoz, A. Structural, Vibrational, and Electronic Study of Sb_2S_3 at High Pressure. *J. Phys. Chem. C* **2016**, *120*, 10547–10558. https://doi.org/10.1021/acs.jpcc.6b01276.
